# Reply to “Rigler’s Triad Another Challenge to Remember”

**DOI:** 10.31662/jmaj.2025-0096

**Published:** 2025-05-26

**Authors:** Ryohei Ono, Izumi Kitagawa

**Affiliations:** 1 Department of Cardiovascular Medicine, Chiba University Graduate School of Medicine, Chiba, Japan; 2 Department of General Internal Medicine, Shonan Fujisawa Tokushukai Hospital, Fujisawa, Japan

**Keywords:** cholelithiasis, diagnosis, Rigler’s triad, treatment

Dear Editor,

We sincerely appreciate the valuable comments from dos Santos VM and Sugai KM regarding our recently published case report on Rigler’s triad ^(1)^. Their interest in this topic and their insightful discussion of additional cases significantly contribute to a deeper understanding of Rigler’s triad, particularly in elderly patients.

A prior review indicated that gallstone ileus is more commonly observed in women, with a female-to-male ratio ranging from 3.5 to 3.6:1, and typically affects individuals over the age of 65 ^(2)^. This aligns with the cases presented by dos Santos VM and Sugai KM, reinforcing the importance of considering Rigler’s triad as a differential diagnosis in elderly patients presenting with small-bowel obstruction. We also agree that prompt recognition and timely surgical intervention are crucial for improving patient outcomes. In general, because the spontaneous passage of gallstones occurs at a low rate of around 1.3%, surgical intervention is recommended for gallstone ileus cases, involving both stone removal to relieve the obstruction and fistula closure ^(3), (4)^. Although our case did not involve closure of the fistula, the persistence of a cholecystointestinal fistula is a potential causal factor for retrograde cholecystitis, gallbladder cancer, or recurrent gallstone ileus ^(3)^.

Furthermore, gallstone ileus develops when an inflamed gallbladder forms adhesions with a neighboring segment of the gastrointestinal tract, leading to pressure-induced necrosis or inflammation that facilitates the passage of gallstones into the bowel. The resulting inflammation or necrosis leads to erosion and the formation of a cholecyst-enteric fistula ^(5)^. Therefore, the predominance of Rigler’s triad in elderly women, as observed in both our case and the additional cases presented, may suggest a potential correlation between age-related changes in gallbladder motility and the development of biliodigestive fistulas leading to gallstone ileus. Further research into these pathophysiological mechanisms may enhance our understanding and improve diagnostic strategies.

Their thoughtful comments contribute to the ongoing discussion on this rare but clinically significant condition, particularly regarding Rigler’s triad. Continued reporting of such cases will help refine diagnostic and therapeutic approaches, ultimately benefiting patient care.

## Article Information

### Conflicts of Interest

None

### Author Contributions

Ryohei Ono: Writing - Original draft

Izumi Kitagawa: Writing - review and editing

All authors approved the final version of the manuscript.

### Approval by Institutional Review Board (IRB)

IRB approval was not required for this study.

### Informed Consent

Informed consent was not required for this study.

## References

[1] Ono R, Kitagawa I. Rigler’s triad: a radiological sign of gallstone ileus. JMA J. 2025;8(1):293-4. 39926058 10.31662/jmaj.2024-0192 PMC11799675

[2] Ploneda-Valencia CF, Gallo-Morales M, Rinchon C, et al. Gallstone ileus: an overview of the literature. Rev Gastroenterol Mex. 2017;82(3):248-54. 28433486 10.1016/j.rgmx.2016.07.006

[3] Inukai K. Gallstone ileus: a review. BMJ Open Gastroenterol. 2019;6(1):e000344. 10.1136/bmjgast-2019-000344 PMC6904169 31875141

[4] Reisner RM, Cohen JR. Gallstone ileus: a review of 1001 reported cases. Am Surg. 1994;60(6):441-6. 8198337

[5] Ravikumar R, Williams JG. The operative management of gallstone ileus. Ann R Coll Surg Engl. 2010;92(4):279-81. 20501012 10.1308/003588410X12664192076377 PMC3025194

